# Decision aid on radioactive iodine treatment for early stage papillary thyroid cancer: update to study protocol with follow-up extension

**DOI:** 10.1186/s13063-015-0819-6

**Published:** 2015-07-14

**Authors:** Anna M. Sawka, Sharon Straus, Gary Rodin, Kevin E. Thorpe, Shereen Ezzat, Amiram Gafni, David P. Goldstein

**Affiliations:** Division of Endocrinology, Department of Medicine, University Health Network, 200 Elizabeth Street, 12 EN-212, Toronto, Ontario Canada, M5G 2C4; Division of Endocrinology, Department of Medicine, University of Toronto, 200 Elizabeth Street, 12 EN-212, Toronto, Ontario Canada, M5G 2C4; Department of Medicine, St Michael’s Hospital and University of Toronto, 30 Bond Street, Shuter 2-026, Toronto, Ontario Canada, M5B 1W8; Department of Psychosocial Oncology, University Health Network and University of Toronto, 16th Floor Room 724, 610 University Avenue, Toronto, Ontario Canada, M5G 2M9; Department of Psychiatry and Palliative Care, University Health Network and University of Toronto, 16th Floor Room 724, 610 University Avenue, Toronto, Ontario Canada, M5G 2M9; Keenan Research Centre, Li Ka Shing Knowledge Institute of St Michael’s Hospital and the Dalla Lana School of Public Health, University of Toronto, 250 Yonge St., 6th Floor, Toronto, Ontario Canada, M5B 1M8; Department of Clinical Epidemiology and Biostatistics, McMaster University, 1280 Main Street West, CRL-208, Hamilton, Ontario Canada, L8S 4K1; Department of Otolaryngology Head and Neck Surgery, University Health Network and University of Toronto, Wharton Head and Neck Centre 3-952, 610 University Avenue, Toronto, Ontario Canada, M5G 2M9

**Keywords:** Papillary thyroid cancer, Decision aid, Knowledge translation, Patient satisfaction

## Abstract

**Background:**

Patient decision aids (P-DAs) are used to inform patients about healthcare choices, but there is limited knowledge about their longer term effects, beyond the time period of decision-making.

**Methods/Design:**

We developed a computerized P-DA that explains the choice of radioactive iodine (RAI) adjuvant treatment or no RAI, for patients with low risk papillary thyroid cancer after total thyroidectomy. The original protocol for a randomized controlled trial, comparing the use of the P-DA (with usual care) to usual care alone, has been published in *Trials*http://www.trialsjournal.com/content/11/1/81. We found that P-DA (with usual care) significantly improved patients’ medical knowledge at the time of decision-making (primary outcome) compared to usual care alone (control). In this update, we present the protocol for an extended follow-up study (15 to 23 months post-randomization), including qualitative and quantitative methods. The patient outcomes evaluated using quantitative questionnaires include: the degree to which patients feel well-informed about their RAI treatment choice, decision satisfaction, decision regret, cancer-related worry, mood, and trust in the treating physician. The qualitative component explores the experiences of RAI treatment decision-making, treatment satisfaction, and trial participation in a representative subgroup of patients. Extended follow-up study results will be described for the entire study population, and data will be compared between the P-DA and control groups.

**Result and Conclusion:**

This mixed methods extended follow-up study will provide data on long term outcomes, relating to the use of a computerized P-DA in decision-making about adjuvant RAI treatment in early stage papillary thyroid cancer.

**Discussion:**

Our results are intended to inform future research in this area, particularly relating to long term effects of the use of P-DAs in making healthcare choices.

**Trial registration:**

Clinicaltrials.gov identifier NCT01083550, registered 24 February 2010 and last updated 5 January 2015

## Update

### Background

Papillary thyroid cancer is rapidly increasing in incidence throughout the world [1], with some of the greatest increases in incidence observed in relatively low risk disease that is confined to the thyroid [1–3]. In patients with low risk papillary thyroid cancer treated with total thyroidectomy, the use of adjuvant radioactive iodine (RAI) (remnant ablation) is subject to evidence uncertainty, and thus treatment is individualized [4, 5]. Greater emphasis on incorporating patients into the treatment decision-making process has recently been recommended by thyroid cancer experts [5].

We previously developed a computerized patient decision aid (P-DA) that explains the choice of RAI adjuvant treatment or no RAI, for patients with low risk papillary thyroid cancer after total thyroidectomy. This is the first P-DA in the field of thyroid cancer care. The protocol for the randomized controlled trial, comparing the use of the P-DA (with usual care) to usual care alone has been published in *Trials* [6]. We also previously reported the results of the primary outcome analysis of this trial, indicating that P-DA (with usual care) significantly improved patients’ medical knowledge at the time of decision-making compared to usual care alone [7]. Furthermore, in a secondary analysis, decisional conflict was significantly reduced in the P-DA group compared to the control group, at the time of decision-making [7]. However, the rate of RAI use was not significantly different between groups [7]. In a pre-planned mixed methods analysis examining the process of RAI decision-making from the perspective of patients, treating physicians were the most frequently cited information source upon which the decision-making was based [8]. Furthermore, patients’ perceptions of malignancy threat (current or future), relative to the valuation of negative aspects of the treatment (such as side effects or including uncertainty of treatment benefit), strongly influenced RAI treatment choice [8]. In another pre-planned secondary analysis, in which we explored the relationship between individual information preference and knowledge acquisition in P-DA users, we found that individuals with a high monitoring information preference accessed more detailed information from our P-DA program [9]. However, high monitoring information preference was not significantly associated with increased medical knowledge in P-DA users [9].

In this update, we outline amendments to our protocol, including extending follow-up of study participants, after securing additional research funding. The overall aim of our follow-up study is to explore the longer term impact of a thyroid cancer P-DA, from patients’ perspectives. The findings of this secondary study are intended to generate hypotheses on the long term psychosocial impact of P-DAs in the context of treatment of low risk papillary thyroid cancer, using both quantitative and qualitative research methods.

## Methods

This is a single-center, parallel design, randomized controlled trial, in which 74 adults with low risk papillary thyroid cancer were randomized in a 1:1 fashion (using computerized randomization), to a one-time viewing of a patient-directed computerized P-DA (with usual care), or no P-DA (with usual care) (Clinicaltrials.gov identifier NCT01083550 [6, 7]). Prior to randomization, the study participants, research study personnel, investigators, and treating physicians were blinded to the allocation [6, 7]. However, following randomization (and immediate administration of the P-DA intervention for the P-DA group), the participants, research study personnel, and treating physicians were not blinded. The extended follow-up study is un-blinded. The P-DA includes an explanation of risks, benefits, evidence uncertainties, and follow-up implications of the choice of adjuvant RAI treatment or no adjuvant RAI treatment. In the original trial protocol, the patients were followed up on until the treatment decision was finalized (between six and 12 months) [6, 7]. An amendment occurred in the protocol, after publication of the original design [6], specifically relating to measurement of a secondary outcome of satisfaction in patient participants and physicians, utilizing a proposed modification of the Client Satisfaction Questionnaire-8 (CSQ-8) [10]. We were denied permission from the developer of the CSQ-8 questionnaire to modify the form for our study, so this outcome was not pursued. The amended protocol was approved by the University Health Network Research Ethics Board, Toronto, Canada. (UHN REB study identifier: 09-0986-BE).

**Fig. 1:**
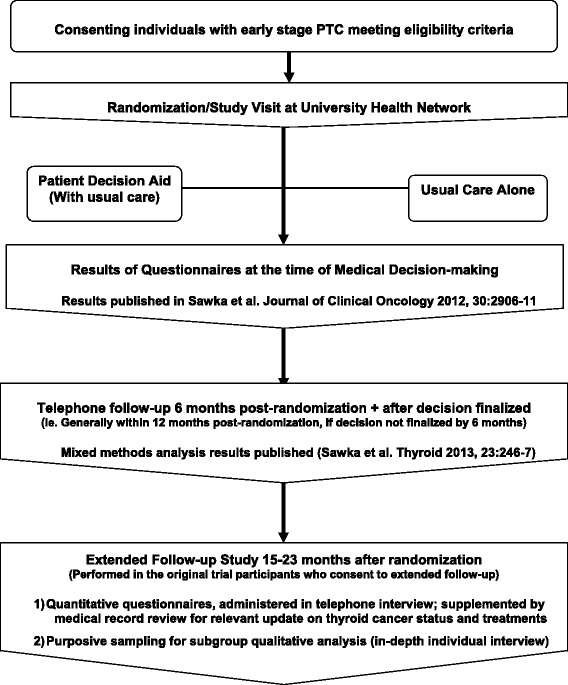
Overview of the study, including update of related publications. For results of questionnaires at the time of medical decision-making and mixed methods analysis results, see Sawka *et al*. [7, 8]. PTC, papillary thyroid cancer; RAI, radioactive iodine

Following initiation of the original trial, we pursued and have been granted additional funding to examine longer term outcomes in our study population. The extended follow-up study has been approved by the University Health Network Research Ethics Board (UHN REB study identifier: 09-0986-BE) and participants provide consent for participation in the study. In this extended follow-up protocol, enrolled randomized participants are contacted by telephone between 15 and 23 months post-randomization, to inquire about interest in participating in a one-time telephone interview, in which demographic and thyroid cancer medical and treatment history are updated and several quantitative questionnaires are administered by phone (Fig. 1). Questionnaires utilized in the telephone interview will include: the degree to which patients feel well-informed about their RAI treatment choice [11], decision satisfaction [11], decision regret [12, 13], cancer-related worry (Assessment of Survivor Concerns) [14], mood [15, 16], and trust in the treating physician [17]. Permission has been sought and granted from developers for the use of all of these questionnaires.

Participants who agree to the phone interview are also asked for consent for relevant medical record review and contact for an in-depth in-person qualitative study (all approved by the University Health Network Research Ethics Board). The medical record review includes collection of information relevant to thyroid cancer disease status, treatment, and follow-up, within the University Health Network electronic medical record or within relevant paper-based records obtained from treating physicians (or the patients themselves), according to the individual patient’s consent, and where their thyroid cancer care is conducted. Patients providing consent for contact of treating physicians are asked to list which physicians may be contacted (including primary care of thyroid cancer specialty physicians or surgeons). Physicians are only contacted for paper-based medical records, and only if consent has been provided from the patient for this purpose. All medical record review data are summarized on a standardized study form by a research staff member and verified by the primary investigator.

In the qualitative component of the extended follow-up study, a representative subgroup of about 15 to 30 consenting participants are invited for an in-person in-depth interview. The criteria used for purposive sampling of participants for the qualitative study include: sex, exposure to the P-DA, and RAI treatment status (that is, having received RAI or not). We strive to achieve equal representation of P-DA users and those not exposed to the P-DA in the qualitative study, and within each of these trial groups, ensuring some representation of those who took RAI as well as those who did not, within each of these study arms. The rationale for ensuring some representation of those taking RAI as well as those not taking RAI within the respective P-DA and non-PDA subgroups, is to enable exploration of the potential effects of P-DA exposure according to the RAI treatment status. In the qualitative study, we strive for a similar sex distribution in both the P-DA and non-P-DA groups, to that observed in the original trial population, largely reflecting the female predisposition to thyroid cancer, but still ensuring some representation from males in both the P-DA and non-PDA groups.

The individual interview is performed by a researcher with experience in qualitative methods, is audio-recorded, and transcribed per verbatim. Interview questions are designed to elicit patients’ perceptions in the following broad areas: the process of RAI treatment decision-making, thyroid cancer treatment satisfaction (and related psychosocial factors), and trial participation. Specifically, participants are asked to discuss the process of how RAI treatment decisions were made, including information sources, and extent of personal involvement in decision-making. Participants are also asked to elaborate on their overall satisfaction with their thyroid cancer care experience, including positive and negative aspects of their experience, any regrets related to the RAI treatment decision, and ongoing worry related to thyroid cancer or its treatment. In addition, participants are asked whether participating in this trial (including exposure to the P-DA or no exposure to the P-DA or any study related follow-up) may have impacted them, and if so, how (for example, influence on RAI-treatment decision-making, thyroid cancer treatment experience, outlook on life, ongoing thyroid cancer-related worry, relationships with the treating physicians or others, or any other way). Participants are also provided the opportunity to provide any open-ended comments about their experiences or the study.

The rationale for selection of the time period of 15 to 23 months post-randomization for this follow-up study, is that it would allow sufficient time for the initial outcome of participants to have been ascertained and communicated to patients from their primary physicians, as well as for resolution of acute RAI treatment side effects (if taken). Furthermore, such a time point is considered close enough to the experience of RAI decision-making that meaningful feedback about the experience could be obtained from participants. This timeline was also chosen given its feasibility, as only one year of funding was approved for the extended follow-up study. All outcomes evaluated in the extended follow-up study are considered secondary and hypothesis-generating.

Demographic and clinical characteristics are summarized for the entire group, as well as for the decision aid and control groups, respectively. Quantitative questionnaires are scored according to instructions from their developers. The quantitative analysis will describe the results of the quantitative questionnaires in the entire study population and both groups, for all individuals consenting to the extended follow-up study. The results of quantitative questionnaires are compared between the P-DA and control groups, using unpaired Student’s t tests. Missing data for any questionnaire subscales is imputed using the mean of the remaining quantitative responses within that subscale (for questionnaires in which subscales are established). An alpha level of 0.05 is established as the cut-off for statistical significance for all comparative analyses, by convention. As the secondary analyses in the extended follow-up study are considered hypothesis-generating, we did not statistically adjust for multiple comparisons, therefore the results will need to be confirmed with future research.

In the qualitative study, purposive participant sampling is continued until saturation of themes is achieved. Content analysis of the qualitative data is completed manually, and the major themes are identified using a grounded theory approach [18–20]. The extracted themes and related quotes are reviewed with a content expert (AMS) for further clarification, as needed.

## Conclusions

There are limited data on the long term impact of P-DAs, as most of the existing trial outcomes have been evaluated at relatively short term time points (that is, one year or less after the healthcare decision) [21]. Thus, this extended follow-up study will provide important insights on longer term outcomes of P-DAs, including important questionnaire outcomes not traditionally studied in the area of P-DAs such as cancer-related worry, physician trust, as well as qualitative data from patients’ perspectives. This study will also inform the importance of future research in long term outcome research for decision support interventions.

## Abbreviations

P-DA: Patient decision aid
RAI: Radioactive iodine

## References

[1] Pellegriti G, Frasca F, Regalbuto C, Squatrito S, Vigneri R (2013). Worldwide increasing incidence of thyroid cancer: update on epidemiology and risk factors. J Cancer Epidemiol..

[2] Chen AY, Jemal A, Ward EM (2009). Increasing incidence of differentiated thyroid cancer in the United States, 1988–2005. Cancer.

[3] Enewold L, Zhu K, Ron E, Marrogi AJ, Stojadinovic A, Peoples GE (2009). Rising thyroid cancer incidence in the United States by demographic and tumor characteristics, 1980–2005. Cancer Epidem Biomar.

[4] Cooper DS, Doherty GM, Haugen BR, Kloos RT, Lee SL, Cooper DS (2009). Revised American Thyroid Association management guidelines for patients with thyroid nodules and differentiated thyroid cancer. Thyroid.

[5] Perros P, Boelaert K, Colley S, Evans C, Evans RM, Gerrard Ba G (2014). Guidelines for the management of thyroid cancer. Clin Endocrinol (Oxf)..

[6] Sawka AM, Straus S, Brierley JD, Tsang RW, Rotstein L, Rodin G (2010). Decision aid on radioactive iodine treatment for early stage papillary thyroid cancer--a randomized controlled trial. Trials..

[7] Sawka AM, Straus S, Rotstein L, Brierley JD, Tsang RW, Asa S (2012). Randomized controlled trial of a computerized decision aid on adjuvant radioactive iodine treatment for patients with early-stage papillary thyroid cancer. J Clin Oncol.

[8] Sawka AM, Rilkoff H, Tsang RW, Brierley JD, Rotstein L, Ezzat S (2013). The rationale of patients with early-stage papillary thyroid cancer for accepting or rejecting radioactive iodine remnant ablation. Thyroid.

[9] Sawka AM, Straus S, Rodin G, Tsang RW, Brierley JD, Rotstein L (2015). Exploring the relationship between patients’ information preference style and knowledge acquisition process in a computerized patient decision aid randomized control trial. BMC Med Inform Decis Mak..

[10] Larsen DL, Attkisson CC, Hargreaves WA, Nguyen TD (1979). Assessment of client/patient satisfaction: development of a general scale. Eval Program Plann..

[11] Martinez LS, Schwartz JS, Freres D, Fraze T, Hornik RC (2009). Patient-clinician information engagement increases treatment decision satisfaction among cancer patients through feeling of being informed. Patient Educ Couns.

[12] O’Connor AM: User Manual – Decision Regret Scale https://decisionaid.ohri.ca/eval_regret.html (1996, updated 2003). Accessed 12 Jan 2015.

[13] Brehaut JC, O’Connor AM, Wood TJ, Hack TF, Siminoff L, Gordon E (2003). Validation of a decision regret scale. Medical Decis Making.

[14] Gotay CC, Pagano IS (2007). Assessment of Survivor Concerns (ASC): a newly proposed brief questionnaire. Health Qual Life Outcomes..

[15] Kroenke K, Spitzer RL, Williams JB, Löwe B (2009). An ultra-brief screening scale for anxiety and depression: the PHQ-4. Psychosomatics.

[16] Löwe B, Wahl I, Rose M, Spitzer C, Glaesmer H, Wingenfeld K (2010). A 4-item measure of depression and anxiety: validation and standardization of the Patient Health Questionnaire-4 (PHQ-4) in the general population. J Affect Disord.

[17] Anderson LA, Dedrick RF (1990). Development of the trust in physician scale: a measure to assess interpersonal trust in patient-physician relationships. Psychol Rep..

[18] Glaser BG, Strauss AL (1967). The discovery of grounded theory: strategies for qualitative research.

[19] Finlay L (2002). Outing the researcher: the provenance, process, and practice of reflexivity. Qual Health Res..

[20] Denzin LL, Lincoln YS (2005). The Sage handbook of qualitative research.

[21] Stacey D, Légaré F, Col NF, Bennett CL, Barry MJ, Eden KB (2014). Decision aids for people facing health treatment or screening decisions. Cochrane Database Syst Rev..

